# Fast Growth Increases the Selective Advantage of a Mutation Arising Recurrently during Evolution under Metal Limitation

**DOI:** 10.1371/journal.pgen.1000652

**Published:** 2009-09-18

**Authors:** Hsin-Hung Chou, Julia Berthet, Christopher J. Marx

**Affiliations:** Department of Organismic and Evolutionary Biology, Harvard University, Cambridge, Massachusetts, United States of America; Université Paris Descartes, INSERM U571, France

## Abstract

Understanding the evolution of biological systems requires untangling the molecular mechanisms that connect genetic and environmental variations to their physiological consequences. Metal limitation across many environments, ranging from pathogens in the human body to phytoplankton in the oceans, imposes strong selection for improved metal acquisition systems. In this study, we uncovered the genetic and physiological basis of adaptation to metal limitation using experimental populations of *Methylobacterium extorquens* AM1 evolved in metal-deficient growth media. We identified a transposition mutation arising recurrently in 30 of 32 independent populations that utilized methanol as a carbon source, but not in any of the 8 that utilized only succinate. These parallel insertion events increased expression of a novel transporter system that enhanced cobalt uptake. Such ability ensured the production of vitamin B_12_, a cobalt-containing cofactor, to sustain two vitamin B_12_–dependent enzymatic reactions essential to methanol, but not succinate, metabolism. Interestingly, this mutation provided higher selective advantages under genetic backgrounds or incubation temperatures that permit faster growth, indicating growth-rate–dependent epistatic and genotype-by-environment interactions. Our results link beneficial mutations emerging in a metal-limiting environment to their physiological basis in carbon metabolism, suggest that certain molecular features may promote the emergence of parallel mutations, and indicate that the selective advantages of some mutations depend generically upon changes in growth rate that can stem from either genetic or environmental influences.

## Author Summary

Effects of mutations can change under different genetic backgrounds or environmental factors, also known as epistasis and genotype-by-environment interactions (G×E), respectively. Though epistasis and G×E are traditionally treated as distinct phenomena, our study of a beneficial mutation highlights their commonality. This mutation resulted from insertion of the same transposable element upstream of a novel cobalt transport system in 30 of 32 independent populations during evolution in metal-limited media. The resulting increased cobalt uptake provided a selective benefit that depended upon two environmental factors: cobalt limitation and growth substrates whose metabolism requires a particular vitamin B_12_ (which contains cobalt) -dependent biochemical pathway. Furthermore, this mutation exhibited epistatic and G×E interactions with other cellular processes in a generic way, such that its selective advantage increased as cells were able to grow faster. This growth-rate dependence accords with a simple model: the slowest of multiple physiological processes needed for growth exerts the greatest control over an organism's growth rate. It suggests that as growth results from the performance of the entire physiological system, genes or environmental factors that affect distinct physiological processes may thus interact through their convergent effects on growth phenotypes.

## Introduction

Adaptation is a product of genetic modification and natural selection imposed by environmental challenges. A complete understanding of adaptation of biological systems thus requires identification of how selection acts upon organismal traits and mapping adaptive phenotypes to underlying genotypic changes. Experimentally testing the genotype-phenotype association and phenotypic effects of mutations is an ongoing research direction in many fields of biology [1]–[3]. Studies on mutations have shown that genetic interactions (epistasis) are common in biological systems [4]–[7] and fitness effects of beneficial mutations can vary greatly depending on environmental conditions (genotype-by-environment interactions, G×E) [8]–[10]. Many studies of beneficial mutations, however, stop short of elucidating the exact molecular mechanisms connecting genotypic changes to phenotypic adaptation [11]–[13]. The lack of this level of information has rendered prediction of fitness effects, epistasis, and G×E interactions elusive. On the other hand, much of our current knowledge of biological systems has come from studying phenotypes of deleterious gene knockouts. Such approaches have uncovered many gene functions and genetic interactions but provided little information about the quantitative response of biological networks to environmental or genetic perturbations as well as the functional significance of a gene in the context of adaptation. A complementary approach to studying the function and evolution of biological systems, therefore, is to characterize molecular mechanisms through which beneficial mutations alter physiology, and reciprocally, how physiological differences due to genetic backgrounds or environments influence the effects of beneficial mutations.

In recent years, evolution experiments using microorganisms have offered a powerful means to investigate the genetic basis of adaptation [14]. Evolution of experimental populations is often conducted using resource-limiting conditions, a challenge many organisms encounter in nature. One competitive strategy to survive under such a scenario is to enhance resource uptake through transport systems. If physiological acclimation is insufficient to alleviate resource limitation, natural selection can favor mutations that further increase uptake capacity. Phenotypes competent to import resources at low concentrations emerge frequently in microbial populations subjected to evolution under resource limitation [10],[15],[16]. Interestingly, beneficial mutations emerging from evolution experiments often occur repeatedly at particular loci [17]. Phylogenetic and quantitative genetic studies of natural populations have also identified many cases of parallel genetic evolution in both micro- and macroorganisms. Frequent observation of genetic parallelism underlying adaptation suggests that, in addition to environmental factors that confine the direction of phenotypic evolution, certain features of the genetic architecture, such as DNA sequence space, genome structure, and the organization of physiological networks may further constrain the breadth of evolutionary trajectories [18].

Metals are essential but often growth-limiting in nature. They are involved in a wide range of physiological processes, such as stabilizing protein structure, relaying cellular signals, and facilitating catalysis in nearly one-third of enzymes [19],[20]. Their most biologically active forms, free cations, however, are limiting in many ecosystems due to oxidation or complexation with organic or inorganic matter [21],[22]. Metal deficiency has been shown to limit the bioproductivity in marine ecosystems and tropical agricultural systems worldwide [23],[24]. For host-pathogen arms races, both animals and plants secrete ligands to sequester metal cations in body fluids to suppress pathogen proliferation, while pathogens have evolved counter-strategies to snatch metals from these ligand-metal complexes [25]–[27]. Clearly, sophisticated metal transport systems and metal-dependent gene regulation mechanisms represent biological adaptation to maintaining metal homeostasis [28],[29] and emphasize the importance of metal acquisition as a prominent fitness component under metal limitation.

In this study, we examined the genetic and physiological basis of adaptation to metal limitation in experimental populations of *Methylobacterium extorquens* AM1 (hereafter *Methylobacterium*) grown in media that we present here as being metal-deficient. In addition to multi-carbon (multi-C) substrates like succinate, *Methylobacterium* can grow on single-carbon (C_1_) compounds like methanol and serves as a model to dissect and engineer metabolic systems of C_1_-utilizing bacteria [30]. Growth of *Methylobacterium* on methanol and on succinate, however, involves several distinct biochemical pathways, and dramatic differences in global gene expression and metabolic profiles have been observed between these two growth modes [31]–[34]. Experimental evolution of populations of *Methylobacterium* grown for 1500 generations on methanol or succinate revealed tradeoffs during adaptation to these two substrates [35]. These tradeoffs were found to be both asymmetric and variable: methanol-evolved populations consistently showed improvements on both substrates, whereas approximately half of the succinate-evolved populations completely lost the ability to grow on all C_1_ compounds. Unexpectedly, in these experiments examining tradeoffs, as well as those applying experimental evolution to select for improved growth of *Methylobacterium* bearing an engineered metabolic pathway, we later discovered that the growth media used for evolution was metal deficient due to over-chelation by ethylenediaminetetraacetic acid (EDTA) present in the media. Long-term evolution under such conditions led to the emergence of mutants with enhanced metal uptake in experimental populations founded by either genotype. Here we show that adaptation of *Methylobacterium* to metal limitation entailed remarkably parallel transpositions of an insertion sequence (IS) element that increased expression of a novel cobalt transporter system. The selective advantage of improving cobalt uptake was specific to methanol growth under metal limitation and seemed to result from sustaining biosynthesis of vitamin B_12_, a cobalt-containing cofactor, to support two vitamin B_12_-dependent reactions in C_1_ metabolism. Intriguingly, this mutation provided a higher selective advantage in genetic backgrounds or growth conditions that conferred faster growth rates, indicating growth-rate dependent epistatic and G×E interactions. This generic growth-rate dependence suggests that as growth results from the performance of the entire physiological system, genes or environmental factors that affect distinct physiological functions may thus interact through their convergent effects on growth phenotypes.

## Results

### Transposition of ISMex4 Increased Transcription of the Novel *icuAB* Gene Cassette through Its Outward Promoter Activity

**Figure 1:**
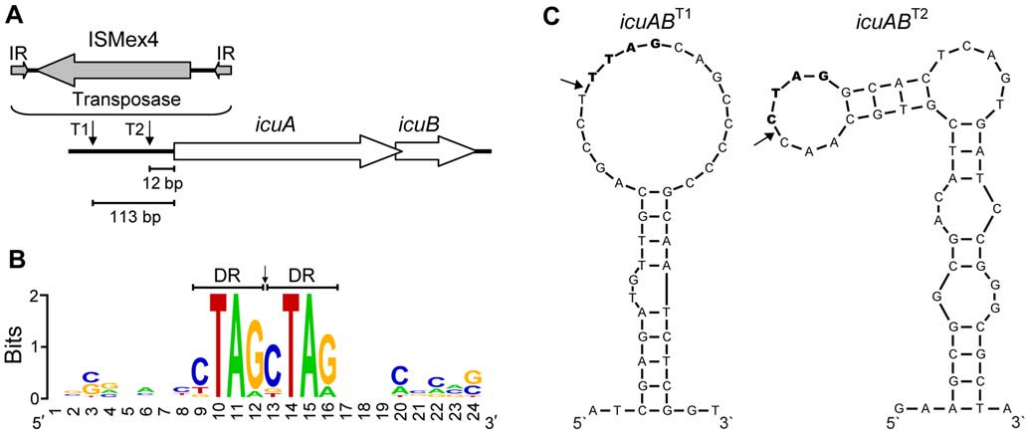
Analysis of transposition sites of ISMex4. (A) Transposition of ISMex4 into two sites upstream of the *icuAB* locus. IR, inverted repeat. T1, Type I insertion. T2, Type II insertion. (B) Conservation of the 4-bp target sequence (or direct repeat, DR) of ISMex4 revealed by alignment of 10 insertion sites. (C) Prediction of the ssDNA structure surrounding ISMex4 insertion sites of *icuAB* ^T1^ and *icuAB* ^T2^ alleles. To deduce the ssDNA structure of original sequences before ISMex4 insertions, the 4-bp direct repeats generated by transposition were removed. Target sequences and insertion sites were indicated by bold text and arrows, respectively.

The IS transposition that occurred across multiple experimental populations was first identified in an evolved isolate, CM1145, from one of the eight methanol-evolving populations (termed F1 to F8) founded by an engineered *Methylobacterium* strain (hereafter termed the EM strain) (Table S1). In the EM strain, the endogenous formaldehyde oxidation pathway required for growth on C_1_ compounds was replaced with a phylogenetically-unrelated formaldehyde oxidation pathway from *Paracoccus denitrificans* [36] (see Plasmid and Strain Construction in the Materials and Methods section). To identify physiological changes that occurred during adaptation of F populations founded with the EM strain, we performed a preliminary microarray analysis to compare genome-wide mRNA pools between the EM strain and the evolved isolate CM1145 (GEO accession no. GSE14875). Further analysis of changes in the transcriptional profile during adaptation in this, and other replicate populations is underway (Chou and Marx, unpublished). Among the observed transcriptional changes from our initial experiment, a putative metal transport cassette increased expression by 50-fold in strain CM1145, relative to the EM strain. Real-time PCR analysis of the two uncharacterized genes in this cassette, *icuA* and *icuB* (**i**mproved **c**obalt **u**ptake phenotype, GenBank accession no. EU679505), revealed 70.8±13.0-fold and 20.0±4.7-fold increased transcription, respectively (throughout we report the mean and 95% confidence intervals based on three replicates). Open reading frames (ORFs) of *icuA* and *icuB* overlap by 4 bp. The *icuA* gene encodes a 704-amino acid protein homologous to TonB-dependent outer membrane receptors. The *icuB* gene encodes a protein of 243 amino acids exhibiting no significant sequence similarity to any characterized gene in public databases. The CD-Search program [37] clustered IcuB with a group of uncharacterized ORFs (CDD accession no. COG5266) predicted to encode periplasmic components of the ABC-type cobalt transport system. PCR amplification of the *icuAB* locus of strain CM1145 detected a 1.6 kb size increase within its 5′ upstream region. Sequencing of the PCR product revealed transposition of an insertion sequence, ISMex4 (GenBank accession no. EU679504), into a site 113 bp upstream of the *icuA* start codon (*icuAB* ^1145^ allele with a ‘Type I’ insertion, thus here *icuAB* ^T1^) (Figure 1A).

**Figure 2:**
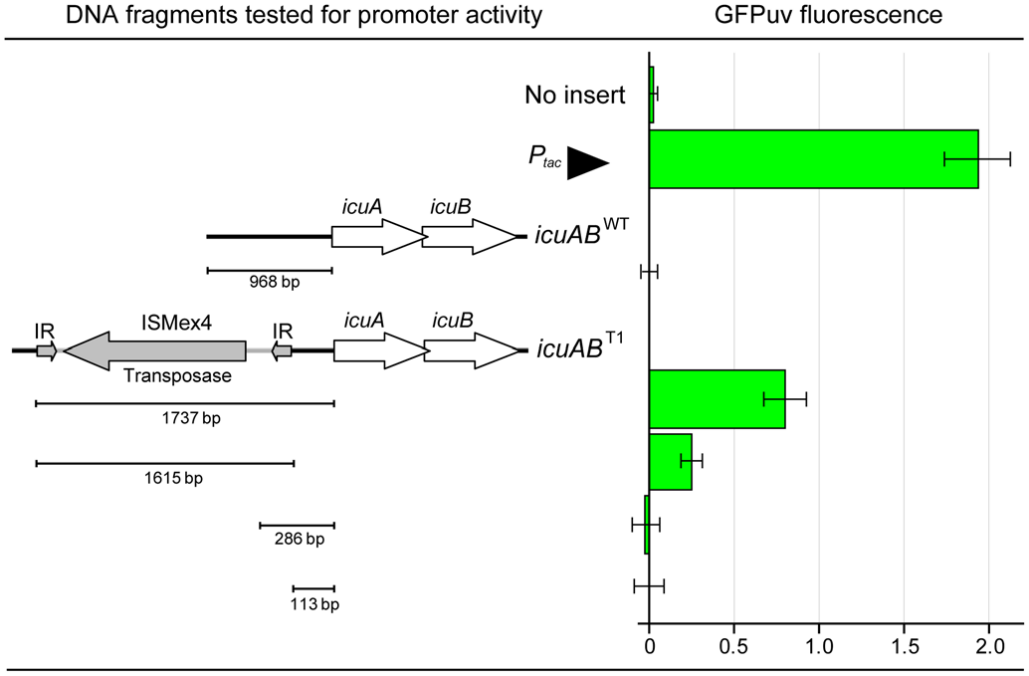
Analysis of promoter activity of the *icuAB* ^WT^ and *icuAB* ^T1^ upstream regions. ISMex4 drives transcription of *icuAB* genes through its outward promoter activity and a synergistic effect between ISMex4 and the adjoining 5′ icuAB upstream region. The fluorescence intensity of GFPuv has been normalized by the cellular autofluorescence (see Fluorescence Measurements in the Materials and Methods section). The bars on the left side of the figure indicate DNA fragments cloned into the promoter probe plasmid to test their promoter activity. Error bars are 95% confidence interval.

Previous studies have shown that transpositions of IS elements may activate transcription of downstream genes by introducing IS-associated outward-directed promoters or by creating hybrid promoters at the junction of insertion [38]. To investigate how ISMex4 insertions enhance transcription of the downstream *icuAB* genes, we measured the promoter activity of the 5′ upstream region of the WT *icuAB* allele (*icuAB* ^WT^) and fragments covering various parts of the *icuAB* ^T1^ 5′ upstream regions with a promoter-probe plasmid using transcriptional fusions to GFPuv (Figure 2). The promoter activity of either a 113-bp or a 968-bp 5′ upstream region of the *icuAB* ^WT^ allele were below the detection limit during growth on methanol. By contrast, ISMex4 alone exhibited significant promoter activity, and the highest activity was observed in the full-length *icuAB* ^T1^ 5′ upstream region (ISMex4 plus the adjacent 113-bp 5′ upstream region). Interestingly, a 282-bp fragment spanning the *icuAB* ^T1^ insertion junction did not exhibit detectable promoter activity. These results suggested that insertion of ISMex4 raised transcription of *icuAB* genes through its outward promoter activity and a synergistic effect between ISMex4 and the adjoining 5′ upstream region, rather than through formation of a hybrid promoter at the insertion junction.

### Transpositions of ISMex4 Occurred Recurrently in Populations Evolved Solely or Partially on Methanol

We used the aforementioned PCR-based screen to survey the *icuAB* locus of evolved isolates across all 8 F populations grown in methanol, as well as the 8 replicate populations each from 4 different evolution experiments founded by the WT strain. These populations, (Table 1, termed A, B, C & D) were grown for 1500 generations on methanol, succinate, both, or alternating between them, respectively [35]. Insertions of ISMex4 into the *icuAB* 5′ upstream region occurred in evolved isolates from 30 out of the 32 A, C, D, and F populations, all of which were evolved solely or partially on methanol. On the contrary, none of isolates from the 8 B populations evolved solely in succinate acquired such mutation. PCR amplification using the 8 B population samples did not detect ISMex4 insertion into the *icuAB* locus among these populations. The pattern of ISMex4 insertions present among A, C, D, F populations versus that of B populations is significantly different (Fisher's exact test, *P*<10^−6^). Sequencing the *icuAB* 5′ region revealed that isolates from 26 populations had an identical ISMex4 insertion as *icuAB* ^T1^. In addition, a second type of ISMex4 insertion was found 12 bp upstream of the *icuA* start codon in strain CM1059 from population C3 (*icuAB* ^1059^ allele with a ‘Type II’ insertion, or *icuAB* ^T2^) and subsequently in four other populations (Figure 1A). This extreme parallelism cannot be accounted for by the presence of these mutations at low frequencies in the ancestral stocks because two types of ancestral genotypes were used in these experiments. In addition, each population was inoculated from a single colony of its respective ancestor. The *icuAB* ^T2^ allele increased transcription of *icuA* and *icuB* by 5.9±0.3 and 6.1±1.4 fold, respectively. For both *icuAB* ^T1^ and *icuAB* ^T2^, the transposase gene of ISMex4 was in inverse orientation to the *icuAB* genes. Sequencing of the *icuAB* 5′ upstream and coding regions of evolved isolates from B populations and the two F populations free of ISMex4 insertion did not identify mutations of any type. ISMex4 has 8 identical copies in the *Methylobacterium* genome [39]. Analysis of these 8 insertion sites along with new insertions identified in this study deduced a 4-bp consensus target sequence (5′-BTAR-3′) that duplicates upon transposition of ISMex4 (Figure 1B) [40]. Analysis by the Mfold program suggested that ISMex4 insertion sites tend to locate in regions prone to form single-strand DNA (ssDNA) secondary structure (Figure 1C and S1) [41].

**Table 1:** Occurrence of ISMex4 insertions among evolved populations.

Pop	Anc	Sub	Gen	Rep	T1	T2	T1&T2	ND
A	WT	M	1500	8	7	1	0	0
B	WT	S	1500	8	0	0	0	8
C	WT	M&S	1500	8	5	3	0	0
D	WT	M/S^a^	1500	8	7	0	1	0
F	EM	M	600	8	6	0	0	2

a Alternating between methanol and succinate each cycle.

Pop, population; Anc, ancestral strain; Sub, substrate; Gen, generation; Rep, number of replicate population; T1, *icuAB* ^T1^; T2, *icuAB* ^T2^; ND, ISMex4 insertion not detected; M, methanol; S, succinate.

### Increased Expression of *icuAB* Improved Cobalt Uptake of *Methylobacterium* in Metal-Deficient Growth Media Due to EDTA Chelation

**Figure 3:**
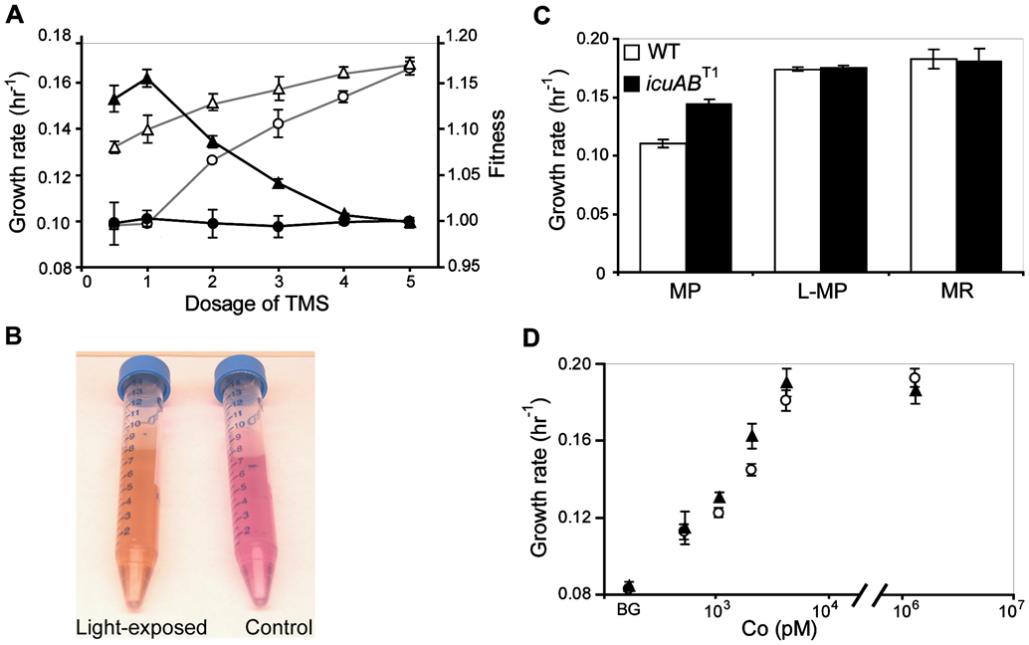
Cobalt is limiting for growth of *Methylobacterium* due to EDTA chelation. (A) Growth rates and fitness values of the *icuAB* ^T1^ mutant (Δ and ▴, respectively) and the WT strain (○ and •, respectively) in response to the dosage of the trace metal solution (TMS). A dosage of 1 corresponds to the amount added to the growth media for the evolution experiments. (B) The color of TMS shifted from purple (control) to orange (light-exposed) over the course of light exposure. (C) Growth rates of the *icuAB* ^T1^ mutant and the WT strain in the metal-poor (MP), light-exposed MP, and metal-rich (MR) media. (D) Growth rates of the *icuAB* ^T1^ mutant (▴) and the WT strain (○) in response to cobalt concentrations in EDTA-free media. BG, background concentration below the detection limit of inductively coupled plasma mass spectroscopy (<0.8 ppb). Error bars are 95% confidence intervals.

To investigate the phenotypes of ISMex4 insertions and the corresponding selection pressure, the *icuAB* ^T1^ or *icuAB* ^T2^ alleles were introduced into WT *Methylobacterium* to replace *icuAB* ^WT^. Since transposition of ISMex4 dramatically elevated transcription of two putative metal-transport genes, we tested whether metal uptake was enhanced by measuring growth rate and fitness of the WT strain and *icuAB* ^T1^ mutant on methanol in media prepared with various doses of trace metal solution (TMS). Growth rate and fitness of the *icuAB* ^T1^ mutant were significantly higher than the WT strain in media prepared with 0.5-, 1- (regular dose), 2-, 3-, and 4-fold TMS, but differences between these two strains diminished with increasing dose, becoming indistinguishable with a 5-fold dose (Figure 3A). The selective advantage of the *icuAB* ^T1^ mutant and its growth rates relative to those of the WT strain were tightly correlated across tested conditions (Pearson's r = 0.990, *P*<0.001). These results indicated: (1) Growth media made with the regular dose of TMS were metal deficient and insufficient to sustain optimal growth of *Methylobacterium* on methanol; (2) Faster growth of the *icuAB* ^T1^ mutant under metal limitation offered a significant competitive advantage.

The observation of poor growth of the WT strain in media with the regular dose of TMS (k = 0.098±0.002) was surprising, given that the growth rate of the same strain at this dose was much higher (k = 0.186±0.003) during the early evolution of these populations. Two observations suggested that the chemical properties of TMS may change upon light exposure: (1) The color of TMS shifted from purple to orange after light exposure (Figure 3B); (2) Growth media made with light-exposed TMS tended to confer faster growth. One potential light-sensitive component in TMS is EDTA, a metal chelator widely applied in growth media to prevent metal precipitation. Previous studies have shown that over-chelation by EDTA can inhibit growth by depleting free metal cations [42],[43]. However, such growth inhibition can be alleviated by exposing media to light, which causes photo-dissociation and photo-degradation of metal-EDTA complexes [44]. We tested if suboptimal growth of *Methylobacterium* in our media resulted from a similar issue. Indeed, the growth rate difference seen above between the WT and *icuAB* ^T1^ mutant vanished in growth media made with light-exposed TMS, consistent with the EDTA over-chelation model (Figure 3C). To ensure the consistency throughout the experiments, TMS and growth media were stored in the dark. Growth media made with the regular dose of TMS were thus termed metal-poor (MP) media. In addition, a different TMS enriched for unchelated metal cations was developed for making metal-rich (MR) media to facilitate the characterization of the Icu phenotype (see Growth Media in the Materials and Methods section). MR media served as a negative control treatment as growth phenotypes of the WT strain and *icuAB* ^T1^ mutant in MR media were indistinguishable from each other (Figure 3C).

As faster growth of the *icuAB* ^T1^ mutant in MP media supported our hypothesis that increased *icuAB* expression enhanced uptake of certain metal species, we tested each of the 7 metals in TMS (Ca, Co, Cu, Fe, Mn, Mo, Zn) to see which one accounted for the beneficial effect. We first measured growth rates of the WT strain and *icuAB* ^T1^ mutant in MP media supplemented with a 3-fold extra dose of EDTA or each of the 7 metal species. While 3-fold extra EDTA completely inhibited growth of both strains, addition of any of the metal species improved growth rates of the *icuAB* ^T1^ mutant (data not shown). Growth rates of the WT strain increased to a smaller extent, and only in response to Co, Fe, Mn, or Zn. These results suggest two possibilities: (1) Growth of *Methylobacterium* in MP media is deficient in all 7 metal species, and overexpression of *icuAB* confers a fitness advantage by enhancing uptake of all of these metals; (2) Addition of any of these metals saturated the metal-chelation capacity of EDTA, resulting in an increase of free metal cations, one (or more) of which was responsible for poor growth and specifically transported by IcuAB. To circumvent the potentially confounding factor of EDTA chelation, we tested growth of the WT strain and *icuAB* ^T1^ mutant on methanol in EDTA-free growth media (see Growth Media in the Materials and Methods section) titrated for the availability of Co, Fe, Mn, or Zn. In the absence of EDTA, only cobalt limitation dramatically slowed growth of both strains. Critically, growth rates of the *icuAB^T1^* mutant were higher than the WT strain at 1.05 (0.062 ppm) and 2.1 (0.124 ppm) nM Co^2+^ (*P*<0.05) (Figure 3D). By contrast, growth responses of both strains were indistinguishable under Fe, Mn, or Zn titration (Figure S2), suggesting that the beneficial effect of IcuAB overexpression likely resulted from improving cobalt uptake.

### The Physiological Basis of Cobalt Requirement for C_1_ Metabolism of *Methylobacterium* Stems from the Ethylmalonyl-CoA Pathway

**Figure 4:**
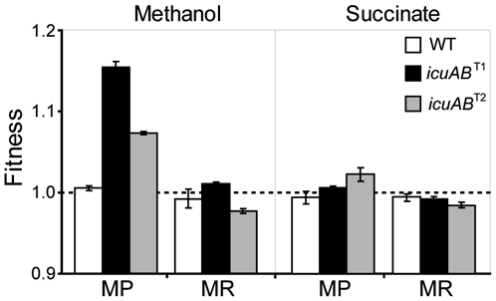
Benefits of the *icuAB* ^T1^ and *icuAB* ^T2^ alleles are specific to methanol growth under metal limitation. Error bars are 95% confidence intervals.

**Figure 5:**
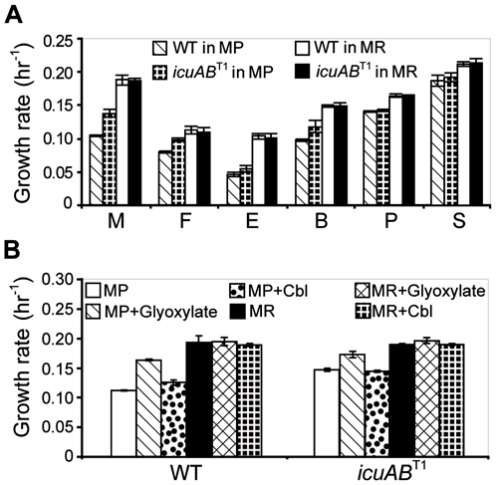
The physiological demand of cobalt stems from AdoCbl-dependent reactions in the EMC pathway. (A) Growth rates of the *icuAB* ^T1^ mutant and the WT strain on various carbon substrates. M, 15 mM methanol; F, 20 mM formate; E, 10 mM ethanol; B, 5 mM betaine, P, 10 mM pyruvate; S, 3.5 mM succinate. (B) Growth rates of the *icuAB* ^T1^ mutant and the WT strain on methanol in MP and MR media supplemented with 1.5 mM glyoxylate or 0.5 mM cobalamin (Cbl). Cyanocobalamin, one type of Cbl, was used as the Cbl supplement. Error bars are 95% confidence intervals.

As ISMex4 transpositions ahead of *icuAB* were nearly universal in populations grown solely or partially on methanol yet were never observed in populations grown solely on succinate, this dichotomy suggested that the advantage of enhancing cobalt uptake came from biochemical reactions specific to methanol (or C_1_) but not succinate (or multi-C) metabolism. Indeed, in MP media the *icuAB* ^T1^ and *icuAB* ^T2^ mutants received higher fitness gains (15.4±0.7% and 7.3±0.2%, respectively) during growth on methanol than on succinate (0.5±0.3% and 2.2±0.8%, respectively) (Figure 4). To identify the responsible biochemical pathway in C_1_,metabolism, we characterized growth phenotypes of the WT strain and *icuAB* ^T1^ mutant on C_1_ (methanol, formate), C_2_ (ethanol), 3C_1_+C_2_ (betaine), C_3_ (pyruvate), and C_4_ (succinate) compounds in MP and MR media. In MR media, growth rates of the WT stain and *icuAB* ^T1^ mutant were indistinguishable on all tested substrates (Figure 5A). In MP media, growth of the *icuAB* ^T1^ mutant was significantly faster than the WT strain only on methanol, formate, ethanol, and betaine (*P*<0.05). As consumption of these four substrates involves metabolism of C_1_ or C_2_ units, this pattern suggests that the demand for cobalt may reside in the overlap of C_1_ and C_2_ metabolism. One such candidate is the ethylmalonyl-CoA (EMC) pathway. This pathway is required to regenerate glyoxylate from acetyl coenzyme A (acetyl-CoA) during growth on C_1_ and C_2_, but not C_3_ and C_4_ compounds of *Methylobacterium* [45]–[47]. Notably, two enzymes in the EMC pathway, methylmalonyl-CoA mutase (MCM) and ethylmalonyl-CoA mutase (ECM), require adenosylcobalamin (AdoCbl, a type of vitamin B_12_) for catalytic function. We thus hypothesized that cobalt limitation may lower production of AdoCbl, which impedes growth of *Methylobacterium* on C_1_ and C_2_ compounds by slowing down regeneration of glyoxylate through the EMC pathway. This hypothesis predicted: (1) Supplementation with glyoxylate, which has been shown to complement mutants defective in the EMC pathway [48],[49], should enhance growth in MP media; (2) Addition of cobalamin (Cbl) should produce similar effects. Indeed, adding glyoxylate to MP media significantly increased the growth rate of the WT strain but to a lesser extent for the *icuAB* ^T1^ mutant (*P*<0.05) (Figure 5B), while adding glyoxylate to MR media did not elevate growth rates of either strain. Furthermore, adding Cbl to MP media increased the growth rate of the WT strain slightly (*P*<0.05) but had no effect on the *icuAB* ^T1^ mutant. Adding Cbl to MR media had no effect on growth of either strain. These results support our hypothesis that shortage of AdoCbl reduces production of glyoxylate via the EMC pathway and thus decelerates C_1_ metabolism of the WT strain under cobalt limitation.

### Overexpression of *icuA* or *icuB* Generates the Icu Phenotype, but Neither Gene Is Essential under Metal Limitation

**Figure 6:**
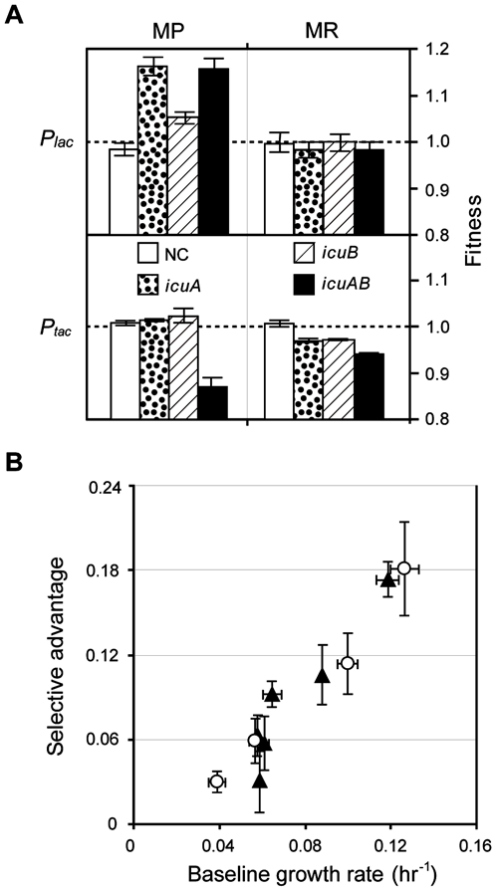
Fitness effects of the *icuAB* gene depend on expression levels, genetic backgrounds, and environmental conditions. (A) Overexpression of *icuA* generates fitness increase similar to the *icuAB* ^T1^ mutant in MP media. (B) The selective advantage of the *icuAB* ^T1^ allele increases with baseline growth rates. Selective advantages of *icuAB* ^T1^ relative to the *icuAB* ^WT^ allele in MP media were measured in a panel of genetic backgrounds grown at 30°C (▴) or in the WT genetic background grown at 16, 20, 25, and 30°C (○). The baseline growth rate indicates growth rates of strains carrying the *icuAB* ^WT^ allele. Error bars are 95% confidence intervals.

Insertion of ISMex4 increases expression of the downstream *icuA* and *icuB* genes. To investigate the individual contribution of these two genes to fitness gain for methanol growth under metal limitation, we overexpressed *icuA*, *icuB*, or *icuAB*, at two expression levels using expression plasmids carrying the *P_lac_* and *P_tac_* promoters, respectively. The promoter activity of the *P_tac_* promoter is approximately 9-fold higher than the *P_lac_* promoter [50],[51]. In MP media, overexpression of *icuA*, *icuB*, and *icuAB* by the *P_lac_* promoter conferred 16%, 5%, and 16% fitness increases (Figure 6A). Overexpression of the *icuA* and *icuB* by the *P_tac_* promoter provided 1% and 2% fitness increases, respectively. Notably, overexpression of *icuAB* by the *P_tac_* promoter incurred a 13% fitness cost under the same growth condition. As overexpression of membrane proteins is often toxic to the organism [52], the negative impact of expressing *icuAB* genes at a higher level may result when its cost exceeds the benefit. In MR media, overexpression of *icuA*, *icuB*, and *icuAB* by the *P_lac_* promoter conferred no benefit and became deleterious when being expressed by the *P_tac_* promoter. Collectively, these results suggest: (1) Overexpression of *icuA* is sufficient to produce a fitness gain similar to the *icuAB* ^T1^ allele; (2) An intermediate optimal expression level exists for the *icuA*, *icuB*, and *icuAB* genes; (3) Expression of *icuA* or *icuB* alone by the *P_tac_* promoter provided positive selective advantages; however, when these two genes were co-expressed by the same promoter, the sum of fitness effect became negative, indicating a negative sign epistasis [53].

To investigate the functional essentiality of the *icuAB* gene cassette, we characterized the phenotypes of Δ*icuA*, Δ*icuB*, and Δ*icuAB* strains grown on methanol or succinate in MP or MR media. Deletion of the *icuAB* cassette was close to selectively neutral (1.006±0.005) during growth on methanol in MR media but resulted in 1.6±0.4%, 1.8±0.7%, and 1±0.1% fitness loss during growth on methanol in MP media, on succinate in MP media, and on succinate in MR media, respectively (Figure S3). Results suggest that *Methylobacterium* possesses alternative systems to uptake cobalt.

### Selective Advantage of the *icuAB* ^T1^ Allele Increases with Growth Rate

In the WT genetic background, acquiring the *icuAB* ^T1^ allele increased growth rate on methanol by 30% in MP media, but introducing this allele to replace *icuAB* ^WT^ of the EM strain did not increase its growth rate under the same growth condition (k = 0.061±0.002 and 0.062±0.004, respectively). In addition, growth rates of the EM strain on methanol in MP and MR media were indistinguishable (k = 0.063±0.001). As the *icuAB* ^T1^ allele emerged in 6 of 8 F populations, these findings raised two questions: (1) Why did the *icuAB* ^T1^ allele exert no detectable effect on growth rate in MP media in the EM genetic background? (2) Why were growth rates of the EM strain in MP and MR media indistinguishable? Growth is a process of biomass assimilation whose rate depends on the rates of multiple resource inputs. A decrease in growth rate may thus weaken advantages conferred by beneficial mutations, like *icuAB* ^T1^, that enhance uptake rates under resource limitation. Since growth of the EM strain was ∼3-fold slower than that of the WT strain, this remarkable difference led us to hypothesize that the selective advantage of the *icuAB* ^T1^ allele may scale generically with the baseline growth rate of the strain. This hypothesis predicts: (1) the selective advantage of the *icuAB* ^T1^ allele should increase when introduced into genetic backgrounds with higher baseline growth rates and (2) the selective advantage should correlate with growth rates modulated by environmental factors independent of cobalt concentrations. First, we measured the fitness effect of the *icuAB* ^T1^ relative to *icuAB* ^WT^ alleles in a panel of genetic backgrounds exhibiting different growth rates: the WT strain, the EM strain, strain CM1145, and three EM-derived strains each bearing individual beneficial mutations found in strain CM1145 (see Plasmid and Strain Construction in the Materials and Methods section). Second, we measured the fitness effect of the *icuAB* ^T1^ allele in the WT genetic background across a range of growth rates resulting from incubation at different temperatures. A potential limitation of this approach is that the genetic and environmental treatments applied undoubtedly modify various phenotypes besides just growth rate, such that each perturbation might display unique interactions with the *icuAB* ^T1^ allele. Intriguingly, the selective advantage of the *icuAB* ^T1^ allele in MP media showed a simple and generic trend: significant positive correlations with the with baseline growth rates across all genetic backgrounds (Pearson's r = 0.940, *P*<0.01) and incubation temperatures (Pearson's r = 0.989, *P*<0.02) (Figure 6B). On the contrary, fitness and growth rates with or without *icuAB* ^T1^ were indistinguishable across all genetic backgrounds and incubation temperatures in MR media where cobalt is not limiting (data not shown). The above results suggest that: (1) the physiological demand on cobalt uptake is higher under faster growth and (2) the selective advantage of the Icu phenotype in MP media may increase as populations adapt toward faster growth.

## Discussion

Despite having been discovered fortuitously, our work represents the first study to investigate the genetic basis of adaptation to metal limitation in an experimental evolution system. As a component of Cbl (or vitamin B_12_), cobalt is critical to biosynthesis of this important coenzyme [54]–[56]. Low concentrations of cobalt in the agricultural and marine ecosystems has been shown to impact human and animal health and reduce vitamin B_12_ production in the ocean, respectively [57],[58]. In this study, evolution under cobalt limitation resulted in emergence of mutants with enhanced cobalt uptake from independent microbial populations. The genetic basis of these independent adaptive events were unusually parallel: resulting from transpositions of ISMex4 into two sites in the *icuAB* 5′ upstream region in 30 of 32 populations grown partial or solely on methanol. On the contrary, such mutation events were never detected in the 8 populations grown solely on succinate. The highly parallel but distinct evolutionary consequences prompted us to investigate the physiological basis of adaptation and molecular features that might promote parallel genetic evolution. We showed that ISMex4 transposition resulted in overexpression of *icuAB* genes, which enhanced cobalt uptake and conferred a substantial fitness increase during growth on methanol in MP media but to a minimal extent on succinate. Our physiological assays further pinpointed the major selective advantage to the need for Cbl in the EMC pathway specifically required for methanol metabolism of *Methylobacterium*, likely resulting from its two AdoCbl-dependent reactions catalyzed by ECM and MCM, respectively. Though the genome sequence suggests two additional Cbl-dependent enzymatic reactions (methione synthase and two ribonucleotide reductases) in *Methylobaterium* [39], the specific growth defect of the WT strain on methanol in MP media and its complementation by glyoxylate support this notion. Like other bacteria, the cytosolic concentration of Cbl in *Methylobacterium* is quite low (∼590 nM) [59]. Cobalt deficiency may thus reduce biosynthesis of Cbl, further lowering its concentration in the cytosol, consequently preventing adenosylation of Cbl and its assembly into ECM and MCM.

Our findings from a laboratory system might have profound implications on how cobalt limitation impacts microbial ecology and evolution in nature. *Methylobacterium* spp. are plant-associated bacteria commonly found on leaves where they compete for nutrients secreted by plants [60]. The ability to utilize methanol, a by-product of plant cell wall synthesis, provides a substantial selective advantage to *Methylobacterium* during epi- and endophytic growth [61]. Nevertheless, the scarce concentration of cobalt (<8 ppb) in plant tissue may pose a difficulty to cobalt transport of *Methylobacterium* as well as other plant-associated bacteria [62]. The importance of cobalt to C_1_ metabolism of *Methylobacterium* makes it compelling to investigate the functional significance of *icuAB* during plant colonization. In fact, cobalt limitation in plants has been demonstrated to inhibit growth and root nodulation of nitrogen-fixing rhizobia [63],[64]. Cobalt may also play a role in the crown gall disease caused by *Agrobacterium tumefaciens*. This pathogen requires indole-3-acetic acid synthesized by a cobalt-containing enzyme to induce abnormal proliferation of plant cells [65]. It would be interesting to apply experimental evolution to study adaptation of plant-associated bacteria in plants grown in cobalt depleted soils. On the other hand, as cobalt is nonessential to plants but essential to many plant microflora [62], it is tempting to ask if the cobalt requirement from plant microflora causes indirect selection on regulation of plant cobalt concentration to welcome mutualistic symbionts or repel harmful pathogens.

On a broader scale, low cobalt concentrations in the environment can greatly impact the supply of vitamin B_12_ to ecosystems as vitamin B_12_ is essential to many organisms but only synthesized by prokaryotes [66]. Across the North Atlantic Ocean, the abundance of phytoplankton and dissolved vitamin B_12_ were found to correlate with cobalt concentrations (0.88−4.77 ppb) [58]. The same study also demonstrated the ability of cobalt to stimulate growth of phytoplankton and vitamin B_12_ production in seawater. *Prochlorococcus* and *Synechococcus*, two dominant photosynthetic bacteria in the open ocean, have an absolute cobalt requirement and appear to secret high-affinity ligands to facilitate cobalt uptake [67],[68]. Combined with genetic and genomic tools, experimental evolution with marine microorganisms represents a promising approach to unravel the genetic and physiological bases of adaptation to metal limitation in the ocean. In addition, the presence of an *icuB* homologue (72% amino acid similarity, Genbank accession no. ZP_00051365) in the genome of the marine magnetotactic bacterium *Magnetospirillum magnetotacticum* MS-1 makes it appealing to address its evolutionary origin and ecological significance.

While environmental and physiological constraints set the stage for the emergence of the Icu phenotype, parallel evolution at the genetic level appeared to be promoted by transposition preference of ISMex4, the chromosomal location of the *icuAB* locus, and clonal interference. In the present study, transposition of ISMex4 conferred a selective advantage by increasing *icuAB* expression, whereas in another study we found an ISMex4 transposition that increased fitness by reducing the transcript level of an overexpressed gene (Chou and Marx, unpublished), stressing the versatile role of IS elements in the evolution of gene expression. Transposition of most IS elements exhibit some degree of target site selectivity [69]. Analysis of ISMex4 insertion sites revealed a 4-bp conserved target sequence that tends to locate in regions prone to form a stem-loop structure. The presence of two ISMex4 copies 15 kb and 38 kb downstream of the *icuAB* genes, respectively, may have increased its chance of transposition into this nearby locus as several IS elements exhibit a high frequency of local-hopping. In addition to aforementioned features that may promote recurrent ISMex4 transpositions, the predominance of the high-fitness *icuAB* ^T1^ allele over the *icuAB* ^T2^ allele across populations suggests a potential role for clonal competition among asexual lineages (Table 1). Identification of two methanol-evolved populations (F5, F6) free of *icuAB* ^T1^ and *icuAB* ^T2^ alleles pointed to the possibility of alternative mutational targets. Compared to growth in MR media, growth rates of evolved isolates from these two populations were just 20% lower in MP media (data not shown), similar to the phenotype of the *icuAB* ^T1^ mutant. Collectively, these results suggest a model shaping genetic parallelism in our system: local-hopping and target selectivity of ISMex4 may lead to high frequency but limited types of transposition while the large fitness advantages gained by *icuAB* ^T1^ and *icuAB* ^T2^ alleles allow them to outcompete other weaker beneficial mutations conferring similar phenotypes in these asexual populations. As the proposed genetic features favoring ISMex4 transposition and its resulting selective advantage can be manipulated easily through mutagenesis and trace metal supplementation, respectively, our system offers the power to experimentally address how mutation rates and the strength of natural selection affect parallel evolution and the dynamics of adaptation.

The physiological effects of an allele depend on expression levels, genetic backgrounds, and environmental conditions. Predicting the behavior and evolution of biological systems requires a comprehensive understanding of how these parameters influence physiology and thus shape the fitness landscape. Experimental evolution offers a valuable alternative besides conventional genetic approaches to uncover biochemical functions and physiological links of genes as well as their contribution to fitness in the evolutionary context. In this study, growth phenotypes of *icuAB* knockout mutants are minute and unspecific to either carbon substrate or growth media, providing no clue to the functional significance of this gene cassette. Nevertheless, by characterizing the phenotypes of beneficial mutations and reconstructing their fitness effect through overexpression experiments, our results revealed the biochemical function of this gene cassette and demonstrated an intermediate optimal expression level that constrains the breadth of phenotypic evolution. Moreover, identification of the physiological processes *icuAB* ^T1^ and *icuAB* ^T2^ contribute to sets the stage to address whether they interact with other mutations or environments in a manner similar to those tested for deleterious mutations. Previous work has shown that growth defects of deleterious mutations tend to be reduced by either environmental stress or the presence of other deleterious mutations [7],[70],[71]. These results have supported a simple model where growth rate is mainly limited by the slowest physiological process [7]. It has remained unclear whether the same principle would apply to certain beneficial mutations, such that they become more advantageous when limitations imposed by other physiological processes are relieved. By modulating growth rate through either incubation temperatures or genetic backgrounds, we found a consistent increase in the selective advantage of a beneficial mutation with increasing growth rate. This growth-rate dependence is in accord with the model described above: By alleviating genetic or environmental constraints, increases in growth rate raised the fitness effect of increased cobalt uptake. The synthesis of previous work with deleterious mutations and current findings from a beneficial mutation suggest a physiology-mediated mechanism through which mutations and environments interact. This mechanism has two profound implications for the evolution and function of biological systems: (1) Some mutations will only be beneficial (or deleterious) when favorable mutations or environmental changes alleviate other physiological limitations, suggesting a general mechanism for historical contingency and environment-dependent evolutionary potential. (2) As higher-order phenotypes (e.g. growth, differentiation, development, locomotion) integrate across multiple physiological inputs, genes and environmental factors that affect seemingly distant physiological processes may thus interact through their convergent effects upon higher-order phenotypic outputs. We anticipate similar observations will continue to emerge from further exploration of the commonality of epistatic, G×E, and even environment-by-environment interactions as flavors of the same phenomenon: systems-wide physiology-mediated interactions.

## Materials and Methods

### Plasmid and Strain Construction

Unmarked allelic exchange plasmids for introducing adaptive mutations or deleting genes were constructed based on pCM433, a *sacB*-based suicide plasmid [72]. Two 3,380-bp PCR fragments containing *icuAB* ^T1^ and *icuAB* ^T2^ alleles were cloned into pCM433 to generate pHC40 and pHC82, respectively (Table S1). The 606-bp and 579-bp PCR products containing *pntA* ^1145^ (encoding membrane-bound transhydrogenase) and *gshA* ^1145^ (encoding γ-glutamylcysteine synthetase) alleles from strain CM1145 were cloned into pCM433 to generate pHC36 and pHC38, respectively. Plasmids pHC65, pHC67, and pHC68 designed to delete *icuA*, *icuB*, and *icuAB*, respectively, were generated by consecutively cloning the 5′ upstream and 3′ downstream regions encompassing the designed deletions into pCM433. A constitutive expression plasmid and a fluorescent promoter-probe plasmid were constructed based on pCM132, a broad-host-range plasmid conferring kanamycin resistance [51]. The *lacZ* gene of pCM132 was deleted and replaced by a 33-bp multiple cloning site to generate pHC41. The promoter-probe plasmid, pHC42, was generated by cloning a 734-bp PCR fragment containing the ribosome binding site (RBS) of the *fae* gene (encoding formaldehyde-activating enzyme) of *Methylobacterium* and the reporter GFPuv gene from pKF133 into pHC41 [73],[74]. A 51-bp synthetic fragment containg the constitutive promoter *P_tac_* was inserted upstream of this reporter to make pHC62. Fragments for testing promoter activity were PCR amplified and inserted into the same cloning site of pHC42 to make pHC44, pHC46, pHC47, pHC51, pHC55. Expression plasmids, pHC60 and pHC91, were generated by cloning the aforementioned 51-bp fragment containing *P_tac_* and a 44-bp synthetic fragment containing the *P_lac_* promoter into pHC41, respectively. PCR fragments containing RBS*_fae_*-*icuA*, RBS*_fae_*-*icuB*, or RBS*_fae_*-*icuAB* were subsequently cloned into pHC60 and pHC91 to generate pHC69, pHC70, pHC71, and pHC92, pHC93, pHC94, respectively.

The EM strain is a variant (Chou and Marx, unpublished) of a previous strain (CM253K.1 with pCM106) shown to be capable of slow growth on methanol [36]. This strain lacks a functional tetrahydromethanopterin-dependent formaldehyde oxidation pathway due to deletion of *mptG* (encoding β-ribofuranosylaminobenzene 5′-phosphate synthase [75]) that eliminated tetrahydromethanopterin biosynthesis. Instead, two genes belonging to the foreign glutathione-dependent formaldehyde oxidation pathway of *Paracoccus denitrificans* (*flhA*, encoding hydroxymethyl-glutathione dehydrogenase and *fghA*, encoding formyl-glutathione hydrolase) were expressed from the strong *P_mxaF_* promoter in plasmid pCM160 [51]. This replacement resulted in restoration of growth on methanol at a one-third the rate of WT [36]. EM-derived strains carrying one of the three other adaptive mutations from strain CM1145 were generated as above. These beneficial mutations affected the expression of aforementioned *fghA*, *pntAB*, and *gshA* genes. Further analysis of the physiological effects of these beneficial mutations will be described subsequently (Chou and Marx, unpublished). The *icuAB* ^WT^ allele was introduced into strain CM1145 by the same allelic exchange method using pHC39. The *icuAB* ^T1^ allele was introduced into the WT strain, the EM strain, and EM-derived strains bearing individual adaptive mutations using pHC40. The *icuAB* ^T2^ allele was introduced into the WT strain using pHC82. Gene knockouts of *icuA*, *icuB*, and *icuAB* were generated by deleting the whole open reading frames from the WT strain. The genotypes of resultant mutants were confirmed by PCR. Strains carrying promoter-probe plasmids or expression plasmids were made by introducing these plasmids from *E. coli* 10-beta (New England Biolabs) into WT *Methylobacterium*, or its isogenic strain CM1180 [35] expressing the yellow fluorescent protein Venus, through tri-parental mating [76].

### Growth Media

The general formula for one liter of all growth media consists of 1 ml of TMS, 100 ml of phosphate buffer (25.3 g of K_2_HPO_4_ and 22.5 g of NaH_2_PO_4_ in 1 liter of deionized water), 100 ml of sulfate solution (5 g of (NH_4_)_2_SO_4_ and 0.98 g of MgSO_4_ in 1 liter of deionized water), 799 ml of deionized water, and the desired carbon sources. One liter of the TMS (pH 5) used in MP media and growth media for evolution experiments consists of 12.738 g of EDTA disodium salt dihydrate, 4.4 g of ZnSO_4_·7H_2_O, 1.466 g of CaCl_2_·2H_2_O, 1.012 g of MnCl_2_·4H_2_O, 0.22 g of (NH_4_)_6_Mo_7_O_24_·4H_2_O, 0.314 g of CuSO_4_·5H_2_O, 0.322 g of CoCl_2_·6H_2_O, and 0.998 g of FeSO_4_·7H_2_O in 1 liter of deionized water [35]. The growth media used for evolution experiments were prepared with this photoactive TMS stored under variable light exposure [35]. MP media were prepared with the same TMS but stored in dark to prevent photochemical reactions. For light exposure experiments, the same TMS was aliquotted into 15 ml plastic tubes (Falcon) covered or uncovered with aluminum foil, then subject to constant light source (broad spectrum, 81 µmol photons m^−2^ s^−1^) for 1 month at 25°C. TMS used in MR media was modified by adding a 4-fold extra dose of iron to displace chelated metals from EDTA-metal complexes [77]. This modified TMS consisted of 10 ml of 179.5 mM FeSO_4_, 80 ml of premixed metal mix (12.738 g of EDTA disodium salt dihydrate, 4.4 g of ZnSO_4_·7H_2_O, 1.466 g of CaCl_2_·2H_2_O, 1.012 g of MnCl_2_·4H_2_O, 0.22 g of (NH_4_)_6_Mo_7_O_24_·4H_2_O, 0.314 g of CuSO_4_·5H_2_O, and 0.322 g of CoCl_2_·6H_2_O in 1 liter of deionized water, pH 5), and 10 ml of deionized water. EDTA-free media were prepared without adding premixed TMS. Instead, each of the 7 trace metal species was supplemented as 0.1 ml of separate solutions (153.02 mM ZnSO_4_, 99.71 mM CaCl_2_, 51.13 mM MnCl_2_, 1.78 mM (NH_4_)_6_Mo_7_O_24_, 12.58 mM CuSO_4_, 13.53 mM CoCl_2_, and 35.9 mM FeSO_4_). Glassware used with EDTA-free media was pre-washed with 0.05 N HCl to eliminate trace metal remnants.

### Evolution Experiments

The A, B, C and D populations were founded by the WT strain [35] while the F populations were founded by the EM strain. All populations were grown in 9.6 ml of growth media contained in 50 ml Erlenmeyer flasks and incubated in a 30°C shaking incubator at 225 rpm. Growth media for evolution experiments consisted of identical minimal growth media supplemented with different carbon sources: A and F populations with 15 mM methanol, B populations with 3.5 mM succinate, C populations with 7.5 mM methanol and 1.75 mM succinate, and D populations alternating between 15 mM methanol and 3.5 mM succinate. The A, B, C and D populations were transferred into fresh growth media at 1/64 dilution rate every two days. Due to the slow growth of the EM strain, the F populations were transferred at the same dilution rate every four days in the first 300 generations of evolution. Transfers of the F populations after generation 300 were switched to two-day cycles. Populations were sampled periodically and preserved at −80°C for later analysis.

### RNA Isolation, Microarrays, and Real-Time PCR

For each strain, three independent mid-exponential phase cultures (defined as half-maximal OD_600_) were harvested and processed by a method described previously [31]. Total RNA was extracted using the RNeasy Mini Kit (QIAGEN), followed by removing residual genomic DNA with the Turbo DNA-free Kit (Ambion). The absence of DNA contamination was verified by PCR amplification of an untranscribed region using primers CM-mxaEdf (5′CTAAGGAAGCCCTGCGATG-3′) and CM-mxaEdr (5′-CCCTCCCGTCTGTTTTTCC-3′). RNA was quantified by a Nanodrop ND-1000 Spectrophotometer (Thermo Scientific) and checked for degradation by an Agilent Bioanalyser 2100 or agarose gel electrophoresis. The preliminary microarray experiment used three independent RNA isolations from each strain that were pooled together with equal quantity. cDNA synthesis, labeling, hybridization to Agilent 60-mer oligo microarrays, and scanning of microarrays were performed by MOgene by following a previously described procedure [31]. cDNA synthesis for real-time PCR was performed using 1 µg total RNA with the qScript cDNA Synthesis Kit (Quanta Biosciences) according to the manufacturer's instructions. The primers used to amplify and detect transcripts of the *icuA*, *icuB*, and *rpsB* genes are HCAM105 (5′-GCTTGCCACCTTCAGCCAGATC-3′) and HCAM106 (5′-ATGGTGACCTTGTTGAAGGCGTTGTA-3′), HCAM107 (5′-TCATCCTCACCGCGCTGC-3′) and HCAM108 (5′-GCTTTGAGCGCGGGCATTG-3′), and HCAM111 (5′-TGACCAACTGGAAGACCATCTCC-3′) and HCAM113 (5′-TTGGTGTCGATCACGAACAGCAG-3′), respectively. Two-step real-time PCR experiments were performed in three replicates with the PerfeCTa SYBR Green SuperMix (Quanta Biosciences) according to the manufacturer's instructions on a DNA Engine Opticon2 (MJ Research). The *rpsB* gene (encoding 30S ribosomal protein S2) was chosen as the reference gene for data normalization. Data analysis was performed with the Opticon Monitor v. 2.02 (MJ Research). The average threshold cycle (Ct) value for each gene was calculated from triplicate reactions for RNA samples by following a previously described method [78]. The ΔCt value described the difference between Ct of the target gene and Ct of the reference *rpsB* gene. The ΔΔCt value described the difference between the ΔCt of the WT strain and that of the evolved or mutant strains. The difference in expression was calculated as 2^ΔΔCt^.

### Detection of IS Insertion and Sequencing

Genomic DNA of 3–6 isolates from each evolved populations was prepared using an alkaline lysis DNA extraction method [79]. The 5′ upstream region of the *icuAB* ^WT^ allele was amplified by primer HCAMp7 (5′-CCGATGGTGAGATCTGGGTCTTCAG-3′) and HCAMp8 (5′-CGTCACCTCCTGACATCTCGATTTAC-3′). Insertions of ISMex4 upstream of the *icuAB* cassette were detected by PCR amplification using primer HCAMp7 and HCAMp38 (5′-ACCAGCACCCGTCCGAGC-3′). The sizes of PCR products were determined by electrophoresis in 1% (w/v) TAE agarose gel. In cases where no PCR product was obtained from sampled isolates, the genomic DNA of the corresponding populations was extracted and PCR amplified through the same means. PCR products of interest were purified with the QIAquick PCR Purification Kit (QIAGEN) and sequenced by MWG Biotech.

### Growth Rate Assays and Competition Experiments

Prior to growth rate assays and competition experiments, all strains were acclimated in growth medium supplemented with carbon sources used in the ensuing assays. Three replicate cultures of each strain were sampled periodically and the change in OD_600_ was measured using a Bio-Rad microplate reader model 680. Competition experiments were performed by following a previously described procedure [35]. Briefly, after one round of acclimation, test strains and a reference strain expressing the yellow fluorescent protein Venus were mixed by a 1∶1 volume ratio, diluted 1∶64 into 9.6 ml of fresh media which were incubated under the conditions described above. The ratios of non-fluorescent cells in mixed populations were measured by passing population samples before (R_0_) and after (R_1_) competition growth through a BD LSR II flow cytometer (BD Biosciences) for at least 50000 cell counts per sample. Fitness values (W) relative to the reference strain were calculated by a previously described equation assuming an average of 64-fold size expansion of mixed populations during competitive growth [35]:
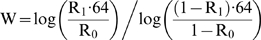



### Fluorescence Measurements

Prior to fluorescence measurements, strains harboring plasmids derived from pHC42 were acclimated in MP media plus 15 mM methanol and 25 µg/ml kanamycin sulfate. Cultures were then grown to exponential phase in the same media without antibiotic. Optical density values at 600 nm (OD_600_) and fluorescence intensities were measured by a Safire^2^ microplate reader (Tecan). The excitation and emission wavelengths for GFPuv were set as 397 nm and 506 nm, respectively [80]. The WT strain was used as control to determine cellular autofluorescence of *Methylobacterium*. To normalize the fluorescence intensity, the fluorescence value of a sample was first divided by its OD_600_. This ratio for the negative control was then subtracted from those of samples to obtain the fluorescence above background. Finally, these values were normalized by dividing the negative control ratio to give the GFPuv fluorescence relative to the background autofluorescence.

## Supporting Information

Figure S1 Prediction of the ssDNA structure surrounding eight original ISMex4 insertion sites in the *Methylobacterium* genome. To deduce the ssDNA structure of original sequences before ISMex4 insertions, ISMex4 and the 4-bp direct repeat generated by transposition were removed. Target sequences and insertion sites are indicated by bold text and arrows, respectively. The META1 numbers indicate the loci where these ISMex4 copies reside on chromosome 1.
(0.32 MB TIF)

Figure S2 The *icuAB* ^T1^ mutant and the WT strain grow similarly in response to iron, manganese, and zinc titration. Growth rates of the *icuAB* ^T1^ mutant (▴) and the WT strain (○) in response to different concentrations of (A) iron, (B) manganese, and (C) zinc in EDTA-free media. Error bars are 95% confidence intervals. BG, undetermined background concentration.
(0.17 MB TIF)

Figure S3 Deletions of *icuA*, *icuB*, and *icuAB* exhibit minor fitness changes in growth on either methanol or succinate.
(0.12 MB TIF)

Table S1 Bacterial strains and plasmids.
(0.11 MB DOC)
