# Supplementary figures and images for: Fast Growth Increases the Selective Advantage of a Mutation Arising Recurrently during Evolution under Metal Limitation
Source: PLoS Genet. 2009 Sep 18;5(9):e1000652. doi: 10.1371/journal.pgen.1000652 (PMC2732905; doi:10.1371/journal.pgen.1000652)

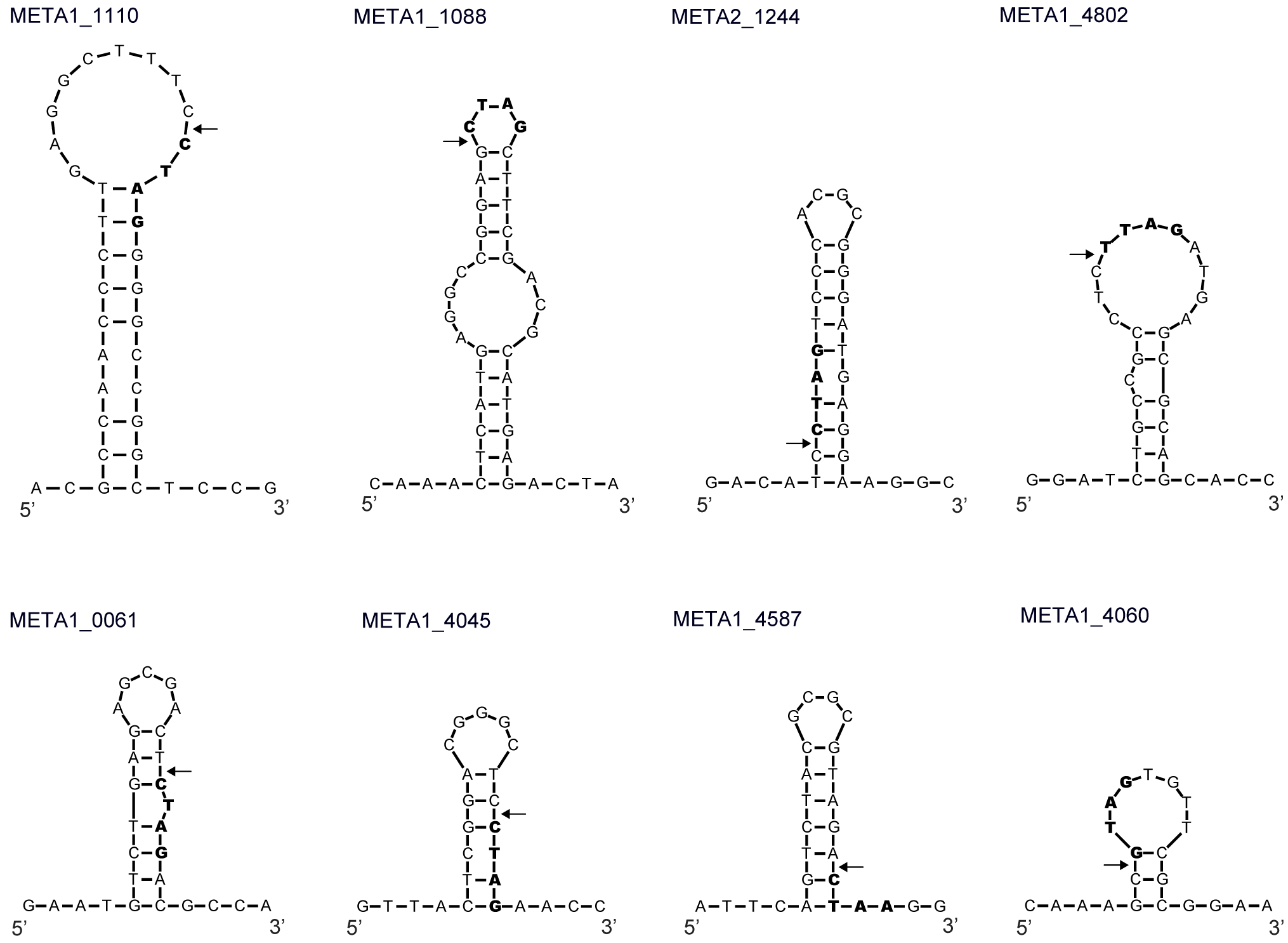

Supplement: Figure S1 — Prediction of the ssDNA structure surrounding eight original ISMex4 insertion sites in the Methylobacterium genome. To deduce the ssDNA structure of original sequences before ISMex4 insertions, ISMex4 and the 4-bp direct repeat generated by transposition were removed. Target sequences and insertion sites are indicated by bold text and arrows, respectively. The META1 numbers indicate the loci where these ISMex4 copies reside on chromosome 1. (0.32 MB TIF) [file pgen.1000652.s001.tif]

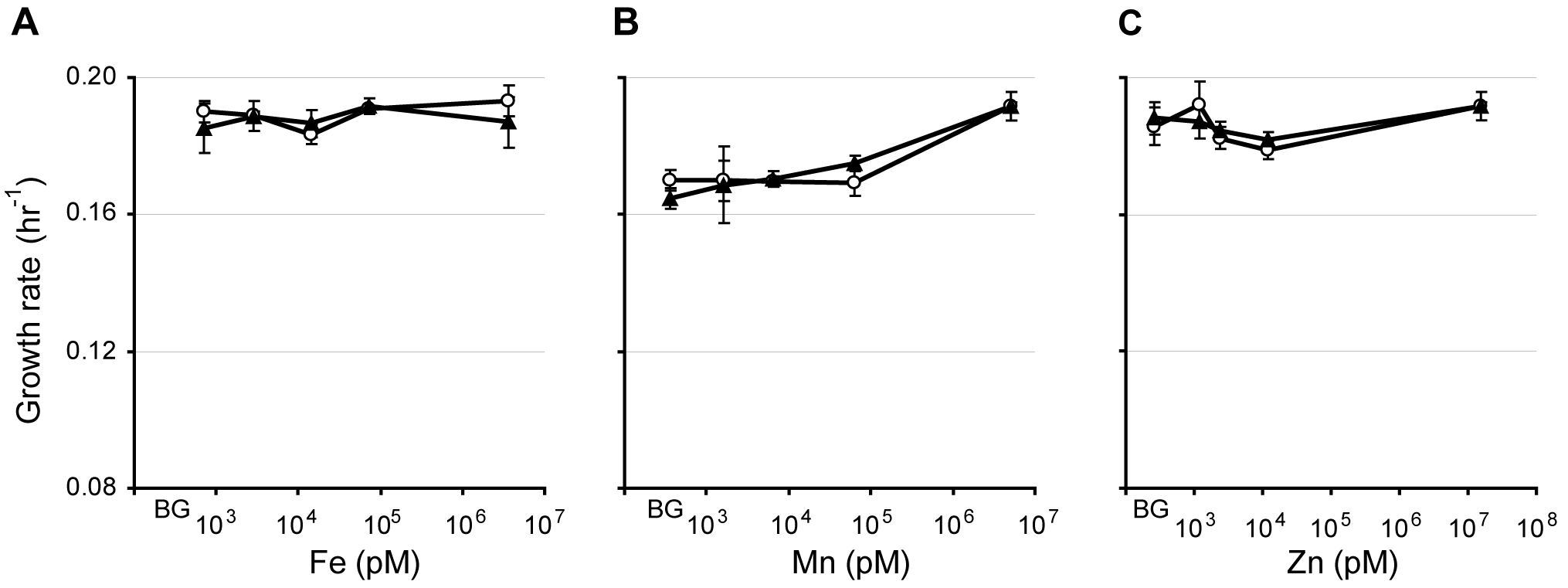

Supplement: Figure S2 — The icuAB T1 mutant and the WT strain grow similarly in response to iron, manganese, and zinc titration. Growth rates of the icuAB T1 mutant (▴) and the WT strain (○) in response to different concentrations of (A) iron, (B) manganese, and (C) zinc in EDTA-free media. Error bars are 95% confidence intervals. BG, undetermined background concentration. (0.17 MB TIF) [file pgen.1000652.s002.tif]

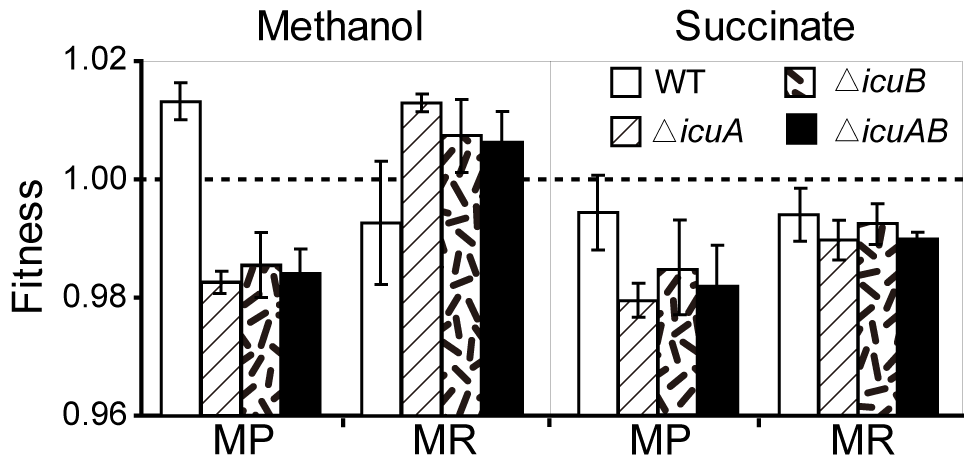

Supplement: Figure S3 — Deletions of icuA, icuB, and icuAB exhibit minor fitness changes in growth on either methanol or succinate. (0.12 MB TIF) [file pgen.1000652.s003.tif]
