# Supplementary material for: Fast Growth Increases the Selective Advantage of a Mutation Arising Recurrently during Evolution under Metal Limitation
Source: PLoS Genet. 2009 Sep 18;5(9):e1000652. doi: 10.1371/journal.pgen.1000652 (PMC2732905; doi:10.1371/journal.pgen.1000652)
Supplement: Table S1 — Bacterial strains and plasmids. (0.11 MB DOC) [file pgen.1000652.s004.doc]

Table S1. Bacterial strains and plasmids

| Strain or plasmid | Description | GenBank accession no. | ^a^Source or reference |
| --- | --- | --- | --- |
| **Strains** |  |  |  |
| WT | Wild-type *Methylobacterium extorquens* AM1 (CM502) |  | 72 |
| EM | Engineered *Methylobacterium* strain (CM702, Δ*mptG*, with pCM410) |  | HC & CJM, unpublished |
| CM1145 | Evolved isolate from the F4 population at generation 600 |  |  |
| CM1180 | WT expressing yellow fluorescent protein Venus |  | 35 |
| CM1275 | EM, *fghA*^CM1145^ |  |  |
| CM1304 | WT, *icuAB*^T1^ |  |  |
| CM1312 | EM, *pntAB*^CM1145^ |  |  |
| CM1316 | EM, *gshA*^CM1145^ |  |  |
| CM1319 | CM1145, *icuAB*^WT^ |  |  |
| CM1320 | EM, *icuAB*^T1^ |  |  |
| CM1321 | EM, *fghA*^CM1145^, *icuAB*^T1^ |  |  |
| CM1792 | EM, *pntAB*^CM1145^, *icuAB*^T1^ |  |  |
| CM1794 | EM, *gshA*^CM1145^, *icuAB*^T1^ |  |  |
| CM1861 | WT, *icuAB*^T2^ |  |  |
| CM1846 | WT, Δ*icuA* |  |  |
| CM1857 | WT, Δ*icuB* |  |  |
| CM1849 | WT, Δ*icuAB* |  |  |
|  |  |  |  |
| **Plasmids** |  |  |  |
| pCM132 | LacZ-based promoter probe plasmid; ^b^Km^r^ | AF327720 | 51 |
| pCM160 | *P_mxaF_* expression plasmid; Km^r^ | AF327717 | 51 |
| pCM433 | *sacB*-based allelic exchange plasmid; ^c^Tc^r^ | EU118176 | 72 |
| pHC36 | pCM433 with *pntAB*^1145^; Tc^r^ |  | HC & CJM, unpublished |
| pHC38 | pCM433 with *gshA*^1145^; Tc^r^ |  | HC & CJM, unpublished |
| pHC40 | pCM433 with *icuAB*^1145^; Tc^r^ | FJ389183 |  |
| pHC41 | pCM132; *lacZ* replaced by a 33-bp polylinker fragment; Km^r^ | FJ389165 |  |
| pHC42 | GFPuv-based Promoter-probe plasmid; Km^r^ | EU679506 |  |
| pHC44 | 968-bp 5` upstream region of *icuAB*^WT^ in pHC42; Km^r^ | FJ389166 |  |
| pHC46 | 282-bp 5` upstream region of *icuAB*^T1^ in pHC42; Km^r^ | FJ389167 |  |
| pHC47 | Full-length ISMex4 in pHC42; Km^r^ | FJ389168 |  |
| pHC51 | 1737-bp 5` upstream region of *icuAB*^T1^ in pHC42; Km^r^ | FJ389169 |  |
| pHC55 | 113-bp 5`upstream region of *icuAB*^WT^ in pHC42; Km^r^ | FJ389170 |  |
| pHC60 | *P_tac_* in pHC41; *P_tac_* expression plasmid; Km^r^ | EU679507 |  |
| pHC62 | *P_tac_* in pHC42; Km^r^ | FJ389171 |  |
| pHC65 | pCM433 with *icuA* upstream and downstream flanks; Tc^r^ | FJ389184 |  |
| pHC67 | pCM433 with *icuB* upstream and downstream flanks; Tc^r^ | FJ389185 |  |
| pHC68 | pCM433 with *icuAB* upstream and downstream flank; Tc^r^ | FJ389186 |  |
| pHC69 | pHC60 with RBS*_fae_*-*icuA* ; Km^r^ | FJ389172 |  |
| pHC70 | pHC60 with RBS*_fae_*-*icuB* ; Km^r^ | FJ389173 |  |
| pHC71 | pHC60 with RBS*_fae_*-*icuAB* ; Km^r^ | FJ389174 |  |
| pHC82 | pCM433 with *pntAB*^CM1059^; Tc^r^ | FJ389187 |  |
| pHC91 | *P_lac_* in pHC41; *P_lac_* expression plasmid; Km^r^ | FJ389176 |  |
| pHC92 | pHC91 with RBS*_fae_*-*icuA* ; Km^r^ | FJ389177 |  |
| pHC93 | pHC91 with RBS*_fae_*-*icuB* ; Km^r^ | FJ389178 |  |
| pHC94 | pHC91 with RBS*_fae_*-*icuAB* ; Km^r^ | FJ389179 |  |

^a^All strains and plasmid from this study unless noted otherwise.

^b^Km^r^, kanamycin resistance.

^c^Tc^r^, tetracycline resistance.
